# Supplementary figures and images for: Differential regulation of cranial and cardiac neural crest by serum response factor and its cofactors
Source: eLife. 2022 Jan 19;11:e75106. doi: 10.7554/eLife.75106 (PMC8806183; doi:10.7554/eLife.75106)

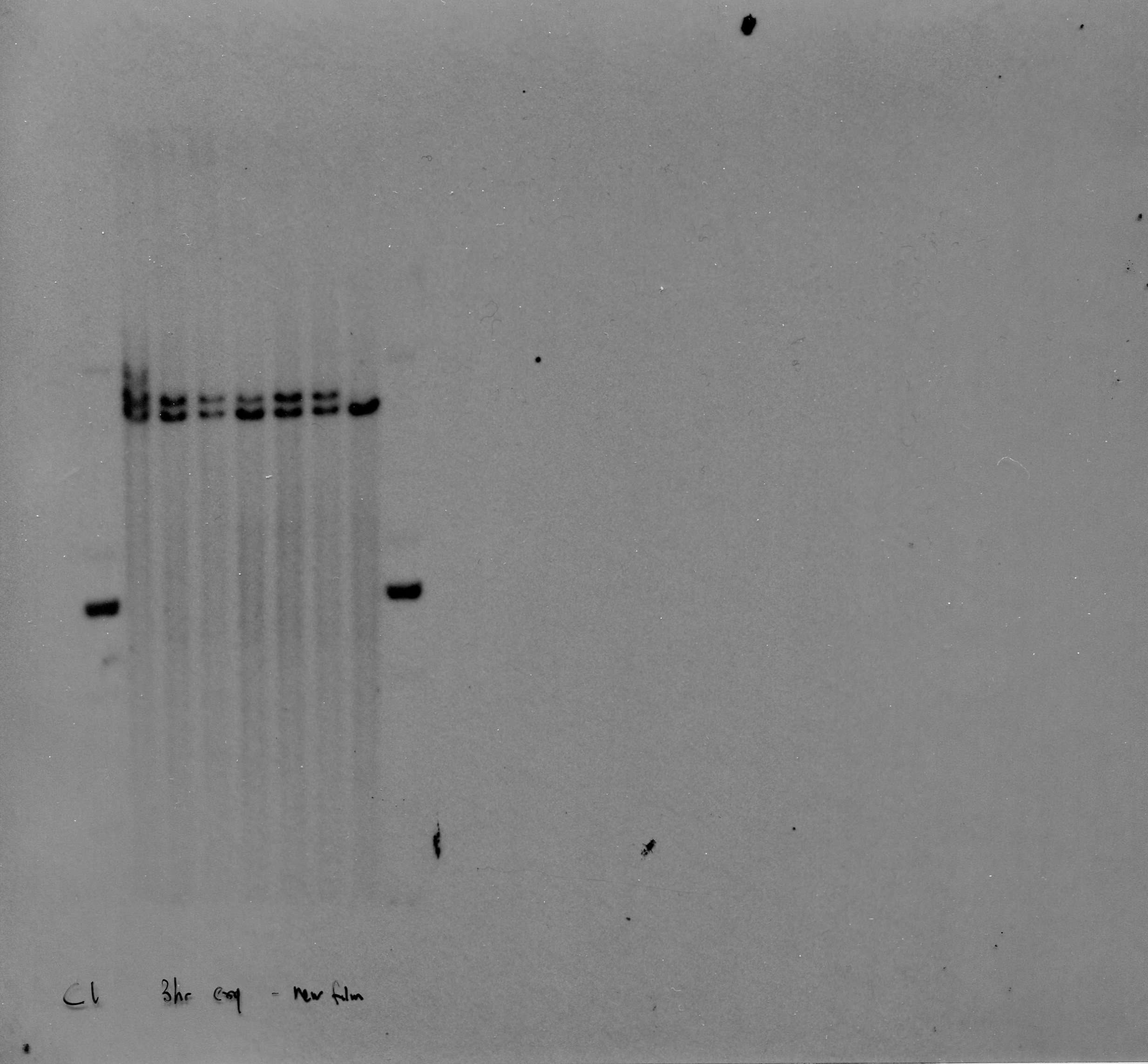

Supplement: Figure 2—figure supplement 1—source data 1. [file elife-75106-fig2-figsupp1-data1.zip › Figure 2 Supplement 1 Source Data 1.jpg]

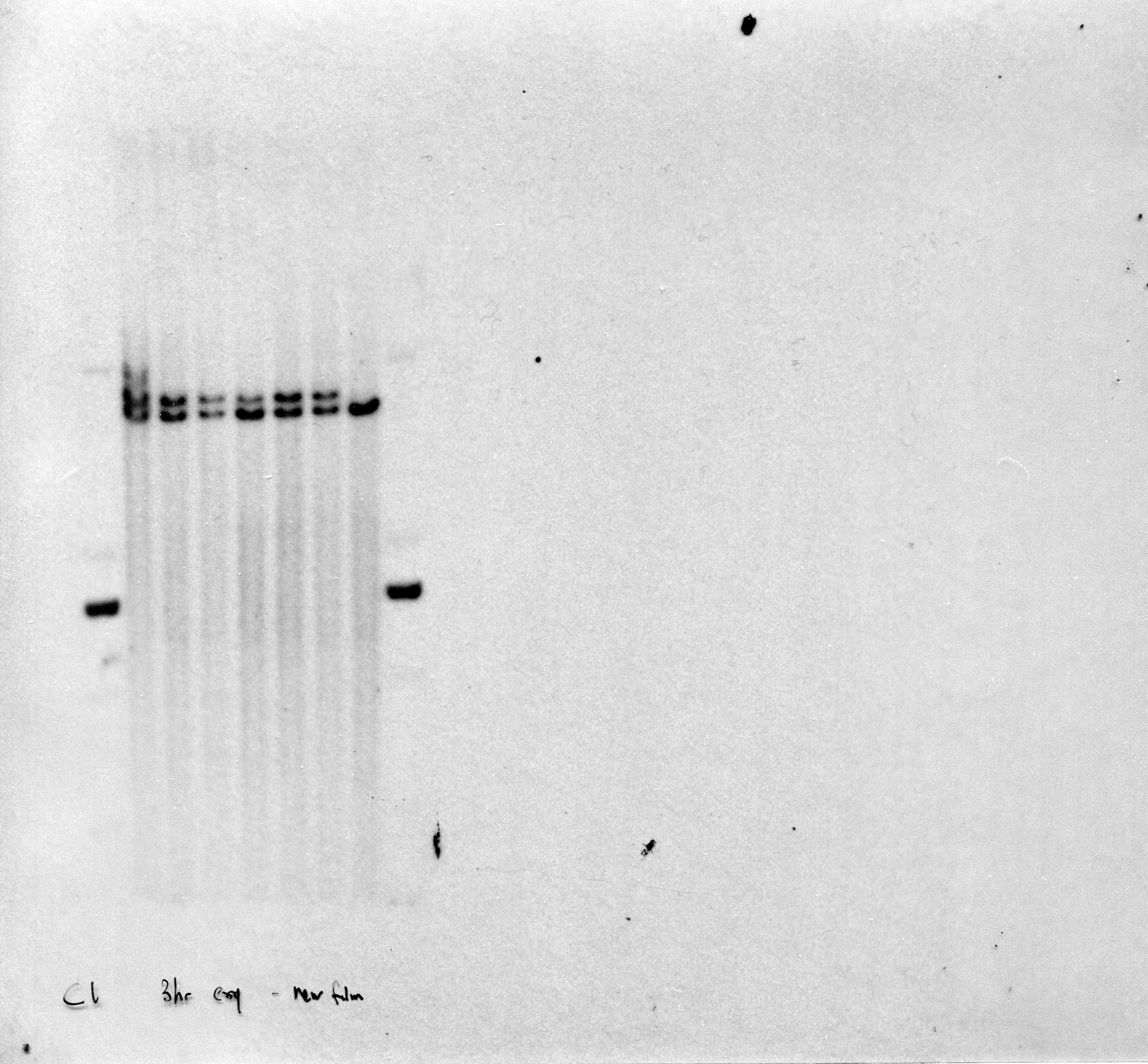

Supplement: Figure 2—figure supplement 1—source data 2. [file elife-75106-fig2-figsupp1-data2.zip › Figure 2 Supplement 1 Source Data 2.tif]
