# Supplementary material for: Differential regulation of cranial and cardiac neural crest by serum response factor and its cofactors
Source: eLife. 2022 Jan 19;11:e75106. doi: 10.7554/eLife.75106 (PMC8806183; doi:10.7554/eLife.75106)
Supplement: Supplementary file 2. — Summary of the mouse knockout phenotypes for Srf and its cofactors, as well as a selection of relevant conditional knockouts discussed in the Introduction and Discussion sections. [file elife-75106-supp2.docx]

**Supplementary file 2. Knockout Phenotypes.**

Summary of the mouse knockout phenotypes for *Srf* and its cofactors, as well as a selection of relevant conditional knockouts discussed in the Introduction and Discussion sections.

| **Genotype** | **Phenotype** | **References** |
| --- | --- | --- |
| Constitutive Knockout Studies | | |
| *Srf^-/-^* | Lethal between E6.5 and E8.5. Fail to induce *T* expression. | (Arsenian et al., 1998; Niu et al., 2005) |
| *Srf^aI/aI^* | Lethal at E10.5. Yolk sac, neural tube, cardiovascular defects. | This Study |
| *Mrtfa^-/-^* | Viable. Females have defect in mammary gland development. | (Li et al., 2006; Y. Sun et al., 2006) |
| *Mrtfb^Gt/Gt^* | Perinatal lethal. Defects in cardiac outflow tract. Rescue with NC-specific transgene. | (J. Li et al., 2005) |
| *Mrtfb^-/-^* | Lethal between E13.5-E15.5. Hemorrhaging and defective cardiovascular development. | (Li et al., 2012; Oh et al., 2005) |
| *Myocd^-/-^* | Lethal from E10.5-E11.5. Cardiovascular defects. Particularly vascular smooth muscle. | (Espinoza-Lewis & Wang, 2014; Li et al., 2003) |
| *Mrtfa^-/-^; Mrtfb^-/-^* | Unknown |  |
| *Elk1^-/-^* | Viable. | (Cesari et al., 2004) |
| *Elk3^-/-^* | Partially viable. Respiratory failure from chylothorax. | (Ayadi et al., 2001; Weinl et al., 2014) |
| *Elk4^-/-^* | Viable, thymocyte positive selection defect. | (Costello et al., 2004) |
| *Elk1^-/-^; Elk3^-/-^; Elk4^-/-^* | Survive until at least E14.5 without morphological defects. Unknown at later stages | (Costello et al., 2010; Gualdrini et al., 2016) |
| Conditional Knockout Studies | | |
| *Srf^flox/flox^; Wnt1-Cre* (Neural crest) | Facial cleft, outflow tract defects, succumbs E13.5-E15.5. | This Study, (Newbern et al., 2008; Vasudevan & Soriano, 2014) |
| *Srf^aI/flox^; Wnt1-Cre* | Succumb postnatally with defects of the cardiac outflow tract. | This Study |
| *Srf^flox/flox^; Mesp1-Cre* (Anterior mesoderm) | Succumb at E10.5 with severe cardiac defects. Undersized, mostly unturned. | This Study |
| *Srf^aI/flox^; Mesp1-Cre* | Succumb at E10.5 with severe cardiac defects. Undersized, partially turned. | This Study |
| *Srf^flox/flox^; Myog-Cre* (Skeletal muscle) | Early postnatal lethality, muscle hypotonia | (S. Li et al., 2005) |
| *Mrtfa^−/−^; Mrtfb^flox/flox^; Myog-Cre* | Similar to above | (Cenik et al., 2016) |
| *Srf^flox/flox^; Nphs2-Cre* (Podocytes) | Podocyte dysfunction including reduced F-actin, structural and gene expression changes, proteinuria | (Guo et al., 2018) |
| *Mrtfa^−/−^; Mrtfb^flox/flox^; Nphs2-Cre* | Similar to above | (Guo et al., 2018) |
| *Srf^flox/flox^; Wt1-Cre* (Epicardium) | Sub-epicardial hemorrhage. | (Trembley et al., 2015) |
| *Mrtfa^−/−^; Mrtfb^flox/flox^; Wt1-Cre* | Sub-epicardial hemorrhage partially due to pericyte reduction. | (Trembley et al., 2015) |
| *Srf^flox/flox^; Pf4-Cre* (Megakaryocytes) | Macrothrombocytopenia. 50% platelet reduction. | (Halene et al., 2010; Smith et al., 2012) |
| *Mrtfa^−/−^; Mrtfb^flox/flox^; Pf4-Cre* | More severe macrothrombocytopenia than above. Distinct gene expression profile from above. | (Smith et al., 2012) |
