# Supplementary material for: Differential regulation of cranial and cardiac neural crest by serum response factor and its cofactors
Source: eLife. 2022 Jan 19;11:e75106. doi: 10.7554/eLife.75106 (PMC8806183; doi:10.7554/eLife.75106)
Supplement: Supplementary file 3. — A list of genotyping primers and product sizes. All reactions were run for 35 cycles with an annealing temperature of 60 ° C. [file elife-75106-supp3.docx]

**Supplementary file 3. Genotyping Primers.**

A list of genotyping primers and product sizes. All reactions were run for 35 cycles with an annealing temperature of 60º C.

| **Reaction** | **Primer 1** | **Primer 2** | **Primer 3** | **Primer 4** | **WT Product** | **Mutant Product** |
| --- | --- | --- | --- | --- | --- | --- |
| *Srf^flox^* | TGCTTACTGGAAAGCTCATGG | TGCTGGTTTGGCATCAACT |  |  | 210 bp | 430 bp |
| *Srf^-^* | TGCTTACTGGAAAGCTCATGG | CTAACCCTGCCTGTCCTTCA |  |  |  | 475 bp |
| *Srf^FLAG^, Srf^aI^* | GATGAACGATGTGACCTCGC | AGGGAGGAGCCAACTCCTTA |  |  | 347 bp | 467 bp |
| *Pdgfra^H2B-EGFP^* | CCCTTGTGGTCATGCCAAAC | GCTTTTGCCTCCATTACACTGG | ACGAAGTTATTAGGTCCCTCGAC |  | 451 bp | 242 bp |
| *Cre* | GCTGCCACGACCAAGTGACAGCAATG | GTAGTTATTCGGATCATCAGCTACAC |  |  |  | 400 bp |
| *MORE-Cre* | GGGACCACCTTCTTTTGGCTTC | AAGATGTGGAGAGTTCGGGGTAG | CCAGATCCTCCTCAGAAATCAGC |  | 411 bp | 311 bp |
| *ROSA26^mTmG^* | CTCTGCTGCCTCCTGGCTTCT | CGAGGCGGATCACAAGCAATA | TCAATGGGCGGGGGTCGTT |  | 330 bp | 250 bp |
| *ROSA26^TdT^* | AAGGGAGCTGCAGTGGAGTA | CCGAAAATCTGTGGGAAGTC | GGCATTAAAGCAGCGTATCC | CTGTTCCTGTACGGCATGG | 297 bp | 196 bp |
