# Supplementary material for: Differential regulation of cranial and cardiac neural crest by serum response factor and its cofactors
Source: eLife. 2022 Jan 19;11:e75106. doi: 10.7554/eLife.75106 (PMC8806183; doi:10.7554/eLife.75106)
Supplement: Supplementary file 4. — Forward and reverse primer sequences used for qPCR experiments in Figure 4. All primers are listed 5’ to 3’. [file elife-75106-supp4.docx]

**Supplementary file 4. qPCR Primers.**

Forward and reverse primer sequences used for qPCR experiments in Figure 4. All primers are listed 5’ to 3’.

| Gene | Forward Primer | Reverse Primer | cDNA amplicon | Genomic amplicon |
| --- | --- | --- | --- | --- |
| *Acta2* | GGCACCACTGAACCCTAAGG | ACAATACCAGTTGTACGTCCAGA | 135 bp | 1822 bp |
| *Actg1* | ATTGTCAATGACGAGTGCGG | CTTACACTGCGCTTCTTGCC | 93 bp | 419 bp |
| *Egr1* | TGGGATAACTCGTCTCCACC | GAGCGAACAACCCTATGAGC | 92 bp | 770 bp |
| *Fos* | TCCTACTACCATTCCCCAGC | TGGCACTAGAGACGGACAGA | 94 bp | 848 bp |
| *Hprt* | TCCTCCTCAGACCGCTTTT | CATAACCTGGTTCATCATCGC | 95 bp | 10935 bp |
| *Srf* | GTGCCACTGGCTTTGAAGA | GCAGGTTGGTGACTGTGAAT | 108 bp | 1875 bp |
| *Tagln* | GACTGCACTTCTCGGCTCAT | CCGAAGCTACTCTCCTTCCA | 100 bp | 4160 bp |
| *Vcl* | TCTGATCCTCAGTGGTCTGAAC | AAAGCCATTCCTGACCTCAC | 103 bp | 41200 bp |
